# Behavioral testing of minipigs transgenic for the Huntington gene—A three-year observational study

**DOI:** 10.1371/journal.pone.0185970

**Published:** 2017-10-09

**Authors:** Verena Schuldenzucker, Robin Schubert, Lisa M. Muratori, Frauke Freisfeld, Lorena Rieke, Tamara Matheis, Sarah Schramke, Jan Motlik, Nicole Kemper, Ute Radespiel, Ralf Reilmann

**Affiliations:** 1 George-Huntington-Institute, Technology-Park, Muenster, Germany; 2 Institute of Zoology, University of Veterinary Medicine Hannover, Hannover, Germany; 3 Department of Physical Therapy, School of Health Technology and Management, Stony Brook University, Stony Brook, New York, United States of America; 4 Department of Clinical Radiology, University of Muenster, Albert-Schweitzer-Campus 1, Muenster, Germany; 5 Institute of Animal Hygiene, Animal Welfare and Farm Animal Behavior, University of Veterinary Medicine Hannover, Hannover, Germany; 6 Laboratory of Cell Regeneration and Plasticity, Institute of Animal Physiology and Genetics, v.v.i., AS CR, Libechov, Czech Republic; 7 Department of Neurodegenerative Diseases and Hertie-Institute for Clinical Brain Research, University of Tuebingen, Tuebingen, Germany; Emory University, UNITED STATES

## Abstract

**Background:**

Large animal models of Huntington’s disease (HD) may increase the reliability of translating preclinical findings to humans. Long live expectancy offers opportunities particularly for disease modifying approaches, but also challenges. The transgenic (tg) HD minipig model assessed in this study exhibits a high genetic homology with humans, similar body weight, and comparable brain structures. To test long-term safety, tolerability, and efficacy of novel therapeutic approaches in this model reliable assessments applicable longitudinally for several years are warranted for all phenotypical domains relevant in HD.

**Objective:**

To investigate whether the tests proposed assessing motor, cognitive and behavioral domains can be applied repetitively over a 3-year period in minipigs with acceptable variability or learning effects and whether tgHD minipigs reveal changes in these domains compared to wildtype (wt) minipigs suggesting the development of an HD phenotype.

**Methods:**

A cohort of 14 tgHD and 18 wt minipigs was followed for three years. Tests applied every six months included a tongue coordination and hurdle test for the motor domain, a color discrimination test for cognition, and a dominance test for assessing behavior. Statistical analyses were performed using repeated ANOVA for longitudinal group comparisons and Wilcoxon-tests for intra-visit differences between tgHD and wt minipigs.

**Results:**

All tests applied demonstrated feasibility, acceptable variance and good consistency during the three-year period. No significant differences between tgHD and wt minipigs were detected suggesting lack of a phenotype before the age of four years.

**Conclusions:**

The assessment battery presented offers measures in all domains relevant for HD and can be applied in long-term phenotyping studies with tgHD minipigs. The observation of this cohort should be continued to explore the timeline of phenotype development and provide information for future interventional studies.

## Introduction

Huntington’s disease (HD) is an autosomal dominant neurodegenerative disorder caused by an extended (≥36) CAG repeat in the Huntingtin (HTT) gene [1], which varies in length between individuals [2]. HD is characterized by motor and cognitive dysfunction as well as behavioral and psychiatric changes [1]. To date, there is no causal therapy for HD [3].

HD animal research has focused on rodent models like the R6/2 mouse [4]. The results have been indispensable for research, but none of the disease modifying treatments suggesting efficacy pre-clinically were successfully translated into humans so far [5]. Large animal (LA) models may have the potential to fill a gap between rodents and humans [6–8] and increase translational reliability, since they have a more similar metabolism, body weight and distribution, and more human like brain volume and anatomy [9]. A large animal model recently developed is the transgenic (tg) HD minipig [9], which has been bred successfully for several generations with stable expression of the transgene.

Previously we proposed an assessment battery for these minipigs that included motor, cognitive and behavioral tests [10]. These were developed to resemble clinical assessments for patients with HD conducted in the Unified Huntington’s Disease Rating Scale (UHDRS) [11,12] and tests established in the quantitative motor (Q-Motor) battery [13] used in biomarker studies [14,15] and clinical trials in HD [16,17].

This longitudinal study was conducted to assess whether the repeated long-term application of these motor, cognitive, and behavioral tests is tolerated, safe and thus feasible in minipigs and whether differences in these domains can be detected between tgHD and wildtype (wt) minipigs during a period of three years.

## Materials and methods

### Experimental animals

**Fig 1:**
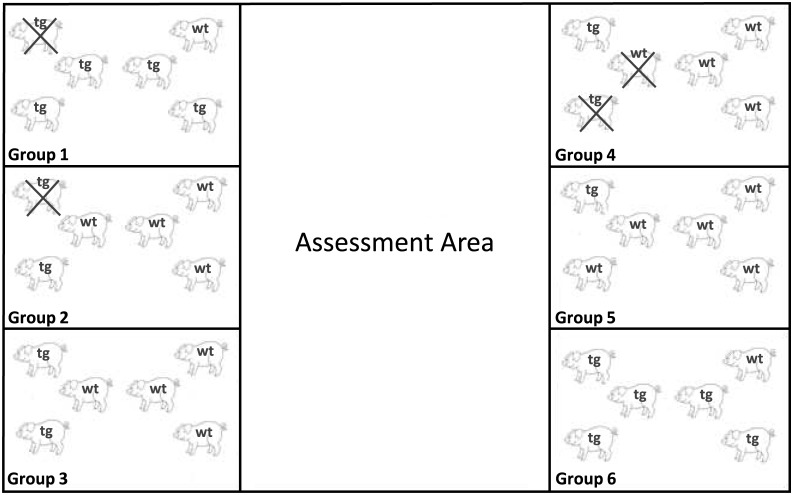
Arrangement of stables. Arrangement of the stables (each~12m^2^) in the ZTE showing all six groups with their individual distribution of genotypes (tg = transgenic, wt = wildtype). The assessment area is located in the middle of the stables and hosts the variable setups outlined in Figs 2 and 3.

The study started with six groups of six female wt and tg (124Q) Libechov minipigs (n = 36, tgHD = 17, wt = 19) with mixed genotypes within each group (Fig 1); the distribution of genotypes in some groups was unequal due to variability in expression of the transgene in different litters. Transgene expression was assessed in fibroblasts of all animals at the beginning of the study after weaning as described before [9]. The Libechov minipig is a mixture of five different races, the Goettinger minipig, Minnesota minipig, Cornwall minipig, the Large Black and Large White minipig. The maximum life expectancy of a Libechov minipig is estimated at 15–20 years. The pigs were bred at the Institute of Physiology and Genetics in Libechov, Czech Republic. Groups arrived in two-month intervals with pigs of around 4 months of age. The central animal facility (ZTE) at the University Hospital of Muenster, Germany, housed the animals in 12m^2^ stables (six animals per stable) with a target temperature of 22°C and a humidity of 50–60% [10]. All animals were provided with toys (e.g. balls and chains), received a special feed two times a day (Wilhelm Reckhorn GmbH & Co. KG, 9091 Minipig Combi and Altromin 9053 special diet for Minipigs) and water ad libitum. A continuous medical surveillance was provided. Weight assessments were performed weekly. The weight of four-year-old female minipigs ranged from 46–128 kg. Two animals (one wt, one tgHD) died due to cardiac events and two tgHD in close proximity to MRI-anesthesia during the three years of follow-up reported here. All statistical analysis reported were performed with the remaining 32 animals (tg = 14, wt = 18), completing the three year follow-up. All study procedures were reviewed and approved by the local governmental animal protection agency prior to initiation of the study.

### Experimental assessment and setup

**Fig 2:**
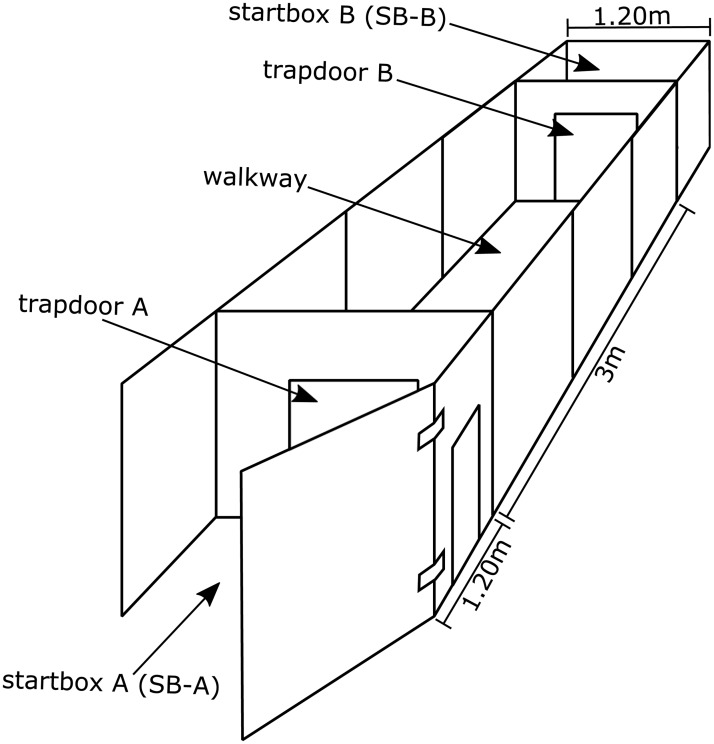
Setup. Basic experimental setup used for the assessment battery, which can be modified to the needs of the individual tests. The startboxes SB-A and SB-B of 1.20m^2^ are located at the end of the 3 m long walkway. The walkway is accessible through trapdoors A and B and holds variable setups as outlined in Fig 3.

All assessments were conducted within the premises of the ZTE. We used a custom-made experimental setup as shown in Fig 2. It was modified for each assessment. All animals performed the test battery twice a year for a total of six sessions covering three years of follow-up time. Each assessment contained defined intervals, which were measured using a stopwatch. At the visit 1(v1) assessment animals were approximately one year old and around four years of age when they completed the sixth visit. For a more detailed description of the assessments listed below refer to our previous report introducing these behavioral methods [10].

### Tongue coordination test (motor domain)

**Fig 3:**
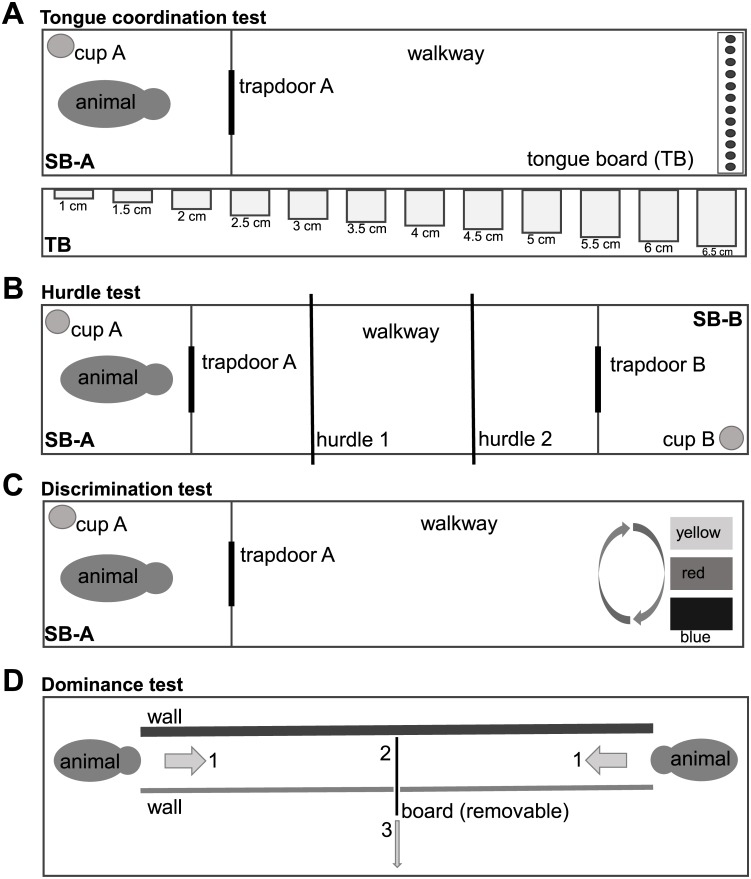
Setup of behavioral tests. (A) In the tongue coordination test, pigs had to enter the walkway and approach the tongue board (TB). The ability to recover the rewards (cornflakes) from holes with continuously increasing depth from left to right was assessed. (B) The hurdle test aimed to assess gait coordination under challenge compared to normal walking. (C) The discrimination test was designed to evaluate the cognitive domain. Minipigs had to explore all boxes and were supposed to learn and remember that only the blue box could be opened. (D) The dominance test was applied to assess behavior. Two animals entered the setup from opposite sides and the animal pushing the opponent backwards was considered dominant. Calculation of an index after exposure of each animal to all group mates was used to determine hierarchy within groups (modified from [27]).

Individuals with HD are incapable of keeping the tongue protruded continuously. This motor impercistency is clinically called “chameleon tongue” and assessed in the tongue protrusion item of the UHDRS-Total Motor Score (UHDRS-TMS). Accordingly, impairment of tongue motor coordination can be detected objectively using the Q-Motor tongue protrusion force analysis (glossomotography) [18]. To assess this feature in minipigs, we developed a tongue function test for pigs [10]. The animals were trained to pick up as many treats (cornflakes) as possible from a board containing 12 holes with stepwise increasing depth from 1–6.5cm. Fig 3A shows the setup of the test and Table 1A the measures recorded. The animals had to complete three consecutive trials on one day at each assessment time—for sample trials see S1 Video.

**Table 1:** Description of measurements.

**A: Tongue coordination test (3 runs)**		
**Measurement**	**Definition**	**Stop times/notes**
Initiation time [s]	Time required to start run by leaving SB-A	Begin: trapdoor A of SB-A is opened End: hip passes trapdoor A while leaving SB-A and entering walkway
Investigation time [s]	Time required to arrive at TB and to initiate investigating it	Begin: minipig is entering walkway End: investigates TB (nose touches TB)
Exploration time [s]	Time required to solve TB task and return to SB-A	Begin: minipig finishes TB task End: hip passes trapdoor A while leaving walkway and reentering SB-A
Depth of holes [cm]	Depth of the deepest hole with successful recovery of treat	Note deepest hole with successful recovery of treat
**B: Hurdle test (6 runs)**		
Initiation time [s]	Time required to start run by leaving SB-A	Begin: trapdoor A of SB-A is opened End: hip passes trapdoor A while leaving SB-A and entering walkway
Run time [s]	Time required to complete run (climbing over hurdles)	Begin: minipig climbs over hurdles End: hip is passing trapdoor B while leaving walkway and entering SB-B
**C: Discrimination test (6 runs)**		
Initiation and exploration time [s]	Time required to start run, pass walkway and open the correct box	Begin: trapdoor A of SB-A is opened and pig’s hip is passing trapdoor A while leaving SB-A and entering walkway End: minipig eats the treats out of the correct box
Attempts to open the yellow/red box	Number of all tries before opening the correct box	Minipig enters walkway and investigates the different boxes
**D: Dominance test (1 run)**		
**CBI = (B+b+1)/(L+l+1)**		
B = number of individuals whom the subject dominates		
b = number of individuals whom those dominated by the subject in turn dominate		
L = number of individuals who dominate the subject		
l = number of individuals who dominate those dominating the subject		

Behavioral tests: Description of the different measurements. (A) Tongue coordination test. (B) Hurdle test. (C) Discrimination test. (D) Dominance Test showing calculation of the Clutton-Brock-Index (CBI). Abbreviations/units: [cm] = centimeters, [s] = seconds, SB = start box, TB = tongue board.

### Hurdle test (motor domain)

In human HD early motor signs can be trigged by balance challenges such as tandem-walking, i.e. walking in a straight line by continuously placing the heel touching the toe, which is assessed in the UHDRS-TMS. Accordingly, we aimed to design a test for minipigs assessing motor function under challenge. We established the hurdle test to serve this purpose [10]. The animals had to climb over two 13-cm-high hurdles—for sample trials see S2 Video. The challenge was repeated six times a day. For a schematic drawing of the setup and a description of the collected measures, see Fig 3B and Table 1B, respectively.

### Discrimination test (cognitive domain)

Cognitive impairments are an important feature of HD [19]. Several tests were developed to assess cognitive signs and symptoms in patients [14,15,20,21]. With regards to assessing cognition in animal experiments, discrimination behavior was successfully tested in HD rodent models [22]. We here present the results of a discrimination test adapted for use in large animals including a protocol for assessing “reversal learning” [10]. The animals had to differentiate between three differently colored (blue, red, yellow) rotating boxes and learn which one could be opened to retreave a treat that was placed in each box—for sample trials see S3 Video. Within the first two test days the blue box was the correct box (6 runs each day). On the next assessment day the yellow box could be opened instead and the pigs had to learn this shift (reversal learning, 6 runs). The setup of the test is shown in Fig 3C and Table 1C describes the measures recorded.

### Dominance test (behavioral domain)

Another aspect of HD are behavioral changes observed during the course of the disease; these include depression, anxiety, irritability, and apathy [15,23,24]. Similar to humans we hypothesised that behavioral changes in animals can be assessed in social interactions. Therefore we designed a “dominance test” for large animals to assess dominant and/or aggressive behavior [10], stimulated by the tube test used in rodents [25,26]. Animals of one group had to encounter each pen mate once every 6 months—for sample trial see S4 Video. We calculated the Clutton-Brock-Index (CBI) for every pig, which quantifies the relative dominance status of each pig in the group [27]. See Fig 3D for the setup and Table 1D detailing the calculation of the CBI.

### Statistics

Statistical analysis was performed using RStudio (Version 3.1.1, R Foundation for Statistical Computing, Vienna, Austria, 2014). To analyze differences between tg and wt pigs longitudinally a 2 (group) x 6 (time) repeated measures ANOVA was conducted for all variables of interest. Within group differences were analyzed with the Wilcoxon-Mann-Whitney Test. This test was also performed, when the ANOVA showed a significant effect of the visit. In this case the difference between visit 1 and visit 6 across groups (n = 32) was tested for exploratory purposes. Significance was set to p≤0.05 (* indicates p≤0.05, ** indicates p≤0.01, *** indicates p≤0.001). No adjustment for multiple testing was performed in this exploratory study. All figures show the mean results and standard deviation (unless specified otherwise) as described for each assessment.

## Results

### Tongue coordination test

**Fig 4:**
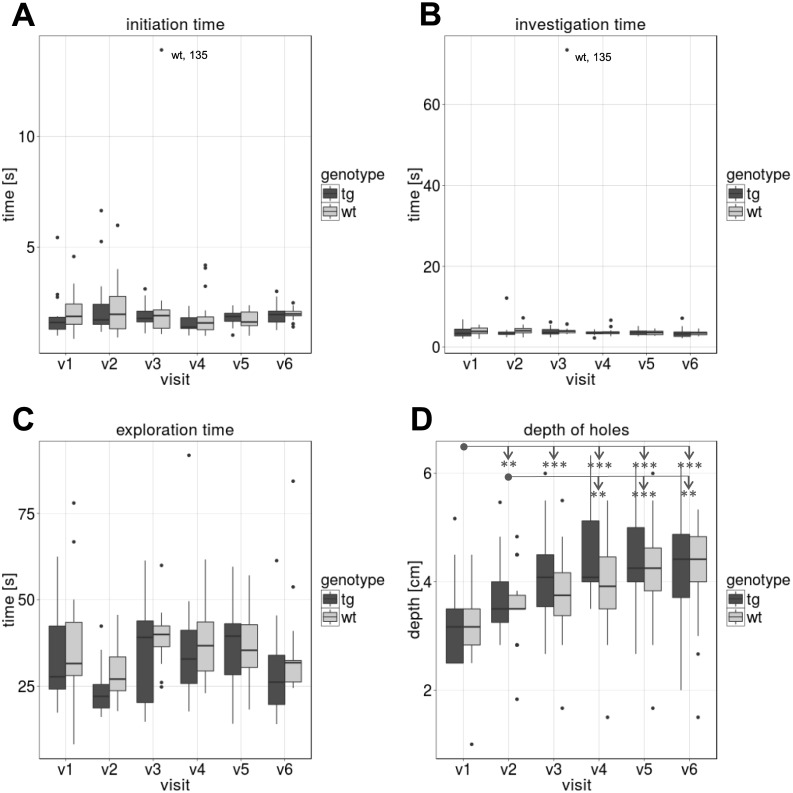
Tongue test. Results of the Tongue coordination test (means of three runs, compared between tgHD and wt minipigs). (A) “Initiation time” i.e. time the pigs need to enter the walkway. (B) “Investigation time”, i.e. time needed to enter the walkway and start investigating (nose contact) the tongue board. (C) “Exploration time”, i.e. time needed to recover all reachable treats and return to the startbox A. (D) “Depth of holes”, i.e. maximal depth recovering treats from tongue board (TB). [* = p≤0.05, ** = p≤0.01, *** = p≤0.001]

Animals were able to learn the Tongue coordination test and reliably completed assessments longitudinally. Fig 4 shows the mean time of three runs for initiation time–Fig 4A, investigation time–Fig 4B and exploration time–Fig 4C; Fig 4D presents the depth of the deepest hole (mean of three runs) the animals reached and succesfully recovered the treat from during the assessment. The animals conducted the tongue test in a consistent way with no significant differences between the tgHD and wt minipigs longitudinally and within each visit. However, Fig 4D shows an overall age effect (p<0.001 in ANOVA, n = 32). Exploratory post-hoc analyses show differences between certain visits across all animals. The data suggests that pigs were able to improve their performance in recovering treats from deeper holes after the first visits.

### Hurdle test

**Fig 5:**
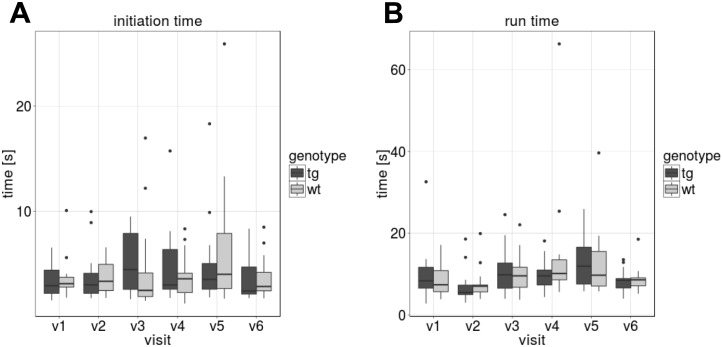
Hurdle test. Results of the Hurdle test (means of six runs, compared between tgHD and wt minipigs per visit). (A) “Initiation time”, i.e. time the pigs need to enter the walkway. (B) “Run time”, i.e. time to complete the walk and arrive in startbox B.

The Hurdle test was feasible to apply and data was collected at all visits. With regards to phenotype and consistency, Fig 5A and 5B show no difference between groups at each visit and no longitudinal changes.

### Discrimination test

**Fig 6:**
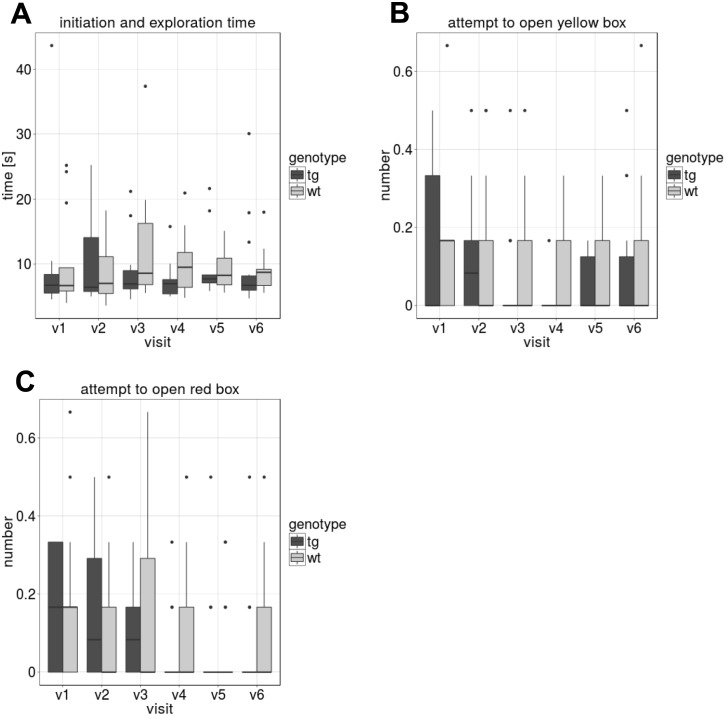
Discrimination test. Results of the blue box Discrimination test at each visit v1 –v6 (means of six runs, compared between tgHD and wt minipigs). (A) “Initiation and exploration time”, i.e. time needed to enter the walkway and open the correct (blue) box. (B) and (C) show the number of attempts to open the red or the yellow box before opening the correct box, respectively.

The discrimination test was successfully established and performed longitudinally. Fig 6 shows the results of the blue box discrimination test. 6A shows the time, the pigs needed to start the test and open the correct (blue) box (initiation and exploration time). There was no difference between tgHD and wt minipigs within the visits and longitudinally. Fig 6B and 6C show the number of times that the minipigs attempted to open the other two incorrect boxes (yellow and red). All attempts prior to opening the correct (blue) box were counted. While the yellow box was the correct one during the next test day (reversal learning), the red box was never correct, which may explain that the number of attempts seemed to decrease towards the last visits—see Fig 6C, albeit this change was not significant.

**Fig 7:**
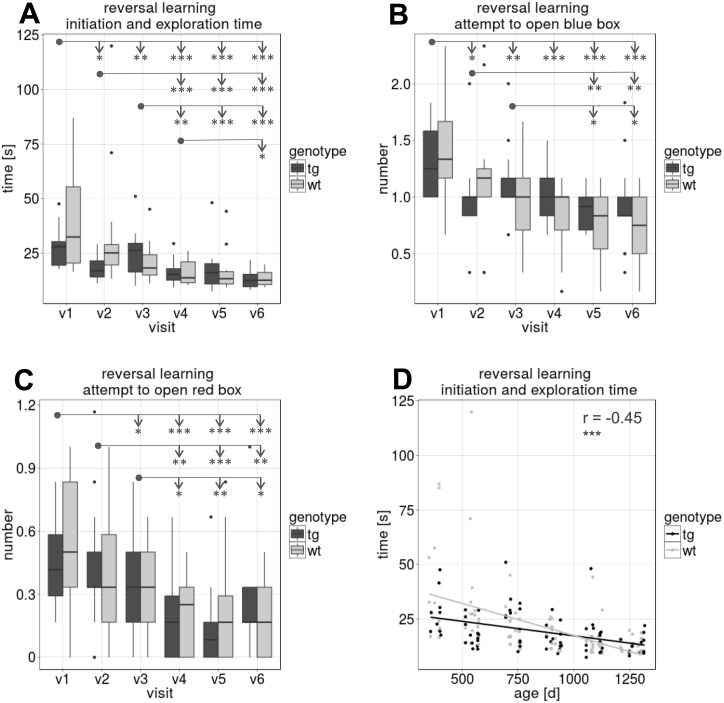
Discrimination test—Reversal learning. Results of the yellow box reversal learning Discrimination test for each visit v1 –v6 (means of six runs, compared between tgHD and wt minipigs). (A) “Initiation and exploration time” needed to enter the walkway and open the correct (yellow) box. Figs (B) and (C) show the number of attempts to open the red or the blue box before opening the correct box. Measures in Figs A-C show a significant decrease in time and number of attempts during the course of the study. (D) Negative correlation between age and “initiation and exploration time” (***). [* = p≤0.05, ** = p≤0.01, *** = p≤0.001]

Fig 7 shows the results of the reversal learning test with the yellow box beeing the correct box to open. The time needed to start and open the yellow box (initiation and exploration time; Fig 7A) and the number of attempts to open the other boxes (blue and red, Fig 7B and 7C) before opening the correct box decreased in the course of the study (p<0.001 in ANOVA, n = 32) which was supported by exploratory post-hoc testing between visits—see arrows indicating between visit differences. Accordingly, Fig 7D shows a negative correlation between the age and the time needed to open the yellow box (r = -0.45, p<0.001, df = 175). No differences between tgHD and wt pigs were seen at any point.

### Dominance test

**Fig 8:**
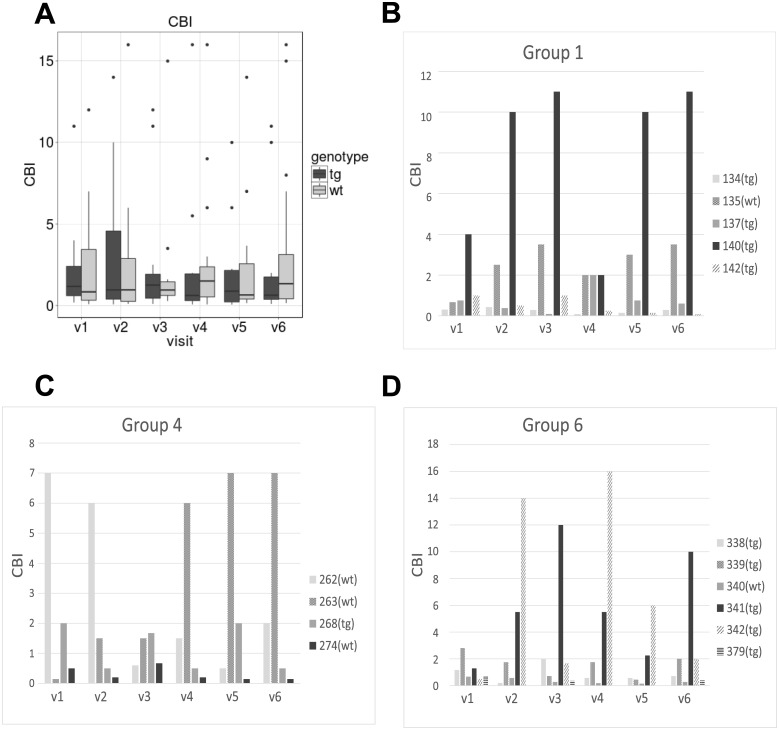
Dominance test. Results of the Dominance test. The figures shows the Clutton-Brock-Index (CBI) [27]. (A) Mean CBI compared between tgHD and wt minipigs at each visit (v1-v6). (B), (C) and (D) sample CBIs of individual pigs in groups 1, 4 and 6 (v1-v6).

The pigs were capable to learn the dominance test and complete all assessments. Fig 8A shows the CBI index of dominance was not different between the tgHD and wt minipigs within any visit or longitudinally. Fig 8B, 8C and 8D show samples of individual CBIs of groups 1, 4, and 6.

## Discussion

This study demonstrates that a complex battery of test assessing motor function, cognition and behavior is applicable and well tolerated in the Libechov transgenic minipig model of HD. The ability of the minipigs to learn all procedures and perform them consistently emphasizes their potential as a large animal model for HD research. While our study revealed no significant difference between the tgHD and wt minipigs during the three-year period assessed, all tests exhibited acceptable variance over time and thus suggest feasibility of consistent task performance for long-term studies.

Therefore, we conclude that the tests introduced can be applied in future studies assessing safety, tolerability and, depending on the target, efficacy of pharmacological and non-pharmacological treatments under development for HD. While lack of symptoms limits the use of the model to assess efficacy of symptomatic therapies, the model can already be used to assess proof-of-concept of, e.g., certain mHTT lowering approaches or distribution pattern, survival and safety of virus vectors in the brain. The long term survival of the model opens the door to a new dimension of safety assessments, which may be of particular value in the context of long term safety of upcoming attempts targeting mHTT lowering in HD or neuronal replacement strategies such as delivery of stem-cells [28]. Another topic of high interest in neurodegenerative diseases is the modulation of neuroinflammation [29], which could be assessed in tgHD minipigs [30] and is target of ongoing clinical trials in HD [31]. Of note, case observations in two older tgHD minipigs suggest manifestation of motor deficits at around 5 years of age (Motlik et al. pers. communication). Thus, further investigations aiming to determine the age of manifestation of symptoms in the domains relevant for HD are warranted and can be achieved applying the battery of tests proposed here. Of note, parts of this battery may also be applicable in other LA models of HD, e.g. sheep, and perhaps translated for research in other neurodegenerative diseases to provide a bridge between rodent models and human clinical trials.

We acknowledge several limitations of the assessment battery. Our results indicate that some tests may be influenced by the animals’ age. Both the tongue and the discrimination test (reversal learning) revealed improved performance with increasing age. In the tongue test this may be explained by the increasing size of the tongue as minipigs grow, which may result in an improved ability to recover treats in deeper holes. The improvement in the reversal discrimination test may indicate a learning effect, similar to what can be seen in human behavioral experiments. The discrimination test may be optimized as successful trials were only counted when pigs retrieved the reward from the designated box. Sometimes the pigs made an attempt at the correct box, but the box moved or turned over preventing successful retrieval of the reward. This resulted in multiple attempts at other boxes and increased time to access the correct box and variability. A setup using fixed retrieval boxes may more accurately assess the pigs’ behavior. In the future, the addition of sensors to quantify measures during tongue protrusion, hurdle crossing and the discrimination tests may improve sensitivity and accuracy and reduce investigator burden. As demonstrated by quantitative motor (Q-Motor) assessments, objective measures increase the sensitivity of assessments in HD patients [13,16]. Similar technologies and automation should improve sensitivity, variability and workload in large animal experiments, as well.

The tgHD minipig model may therefore offer novel opportunities in preclinical research and extend the scope of research established in rodent models of HD [4,31–33]. Minipigs are more comparable to humans in overall body weight and lifespan. For specific investigations, the long lifespan of this model may allow to assess disease processes mirroring the development of HD in humans very closely. The weight range of minipigs (abt. 50-120kg) is similar to the adult humans and offers a good model for pharmacological research. In addition, the size of minipigs and their brain enables detailed MRI scanning, PET acquisition, CSF and blood collection and, e.g., neurosurgical delivery of drugs into the brain using the same tools approved and under development for humans [8]. In terms of genetics and brain structure, LA models exhibit a high similarity to humans (approx. 98.5%). In contrast, the neuroanatomy in rodents is significantly different from humans and the weight of rodent’s brain is about 2g only; minipigs have a structure quite similar to humans with cortical folding and a the weight of the brain is around 90-100g [34–37].

Additional challenges in using large animal models such as minipigs that should be appreciated are the time and costs needed to generate larger number of animals and balance the distribution of tg and wt animals in groups. As genotype distribution in litters varies by chance homogenous distributions are difficult to accomplish. An ethical problem may arise if future interventional studies focus on tgHD animals only, as wt animals will still be generated in roughly equal numbers and issues of animal welfare should be considered proactively. In addition, costs and duration of studies are a relevant factor and present a constraint.

In summary, the long-term applicability of the battery of tests introduced here and the feasibility to generate animals as described supports the conduct of pre-clinical studies in the tgHD minipigs including assessments of motor, cognitive and behavioral domains. Continuation of the current observational study should be considered to assess feasibility of extended long term protocols and inform about possible phenotype manifestation and its course. The knowledge obtained will be important for the design of future pre-clinical studies using the tgHD minipig and possibly more advanced models. It seems conceivable that efforts to generate more advanced genetic minipig models, such as porcine or humanized knock-in models, will be translated rapidly to viable preclinical studies using the methods described.

## Supporting information

S1 Video Tongue coordination test. The video shows two runs of the Tongue coordination test.
(MP4)

S2 Video Hurdle test. The video shows three runs of the Hurdle test.
(MP4)

S3 Video Discrimination test. The video shows three runs of the Discrimination test (blue box correct).
(MP4)

S4 Video Dominance test. The video shows the encounter of two minipigs in the Dominance test.
(MP4)
